# Aminoalkylamides of Eremomycin Exhibit an Improved Antibacterial Activity

**DOI:** 10.3390/ph14040379

**Published:** 2021-04-19

**Authors:** Elena I. Moiseenko, Réka Erdei, Natalia E. Grammatikova, Elena P. Mirchink, Elena B. Isakova, Eleonora R. Pereverzeva, Gyula Batta, Andrey E. Shchekotikhin

**Affiliations:** 1 Gause Institute of New Antibiotics, 119021 Moscow, Russia; moiseenko.alena@gmail.com (E.I.M.); ngrammatikova@yandex.ru (N.E.G.); instna@sovintel.ru (E.P.M.); ebisakova@yandex.ru (E.B.I.); pereverzeva-ella@yandex.ru (E.R.P.); 2 Department of Organic Chemistry, University of Debrecen, Egyetem tér 1, H-4032 Debrecen, Hungary; rerdeii@yahoo.com (R.E.); batta@unideb.hu (G.B.)

**Keywords:** glycopeptide antibiotics, vancomycin, eremomycin, semisynthetic antibiotics, Gram-positive, antibacterial activity

## Abstract

After decades, the glycopeptide vancomycin is still the preferred antibiotic against resistant strains of Gram-positive bacteria. Although its clinical use is strictly regulated, the gradual spread of vancomycin-resistant bacteria, such as glycopeptide-resistant and glycopeptide-intermediate *Staphylococcus aureus* and vancomycin-resistant *Enterococcus* spp., is a serious health problem. Based on the literature data and previous studies, our main goal was to assess the antimicrobial potential and to study the structure–activity relationship of new eremomycin aminoalkylamides. We designed and synthesized a series of new eremomycin amides in which eremomycin is conjugated with a hydrophobic arylalkyl group via an alkylenediamine spacer, and tested their antibacterial activities on a panel of Gram-positive strains that were sensitive and resistant to a “gold-standard” vancomycin. Based on the data obtained, the structure–activity relationships were investigated, and a lead compound was selected for in-depth testing. Research carried out using an in vivo model of staphylococcus sepsis, acute toxicity studies, and the estimated therapeutic index also showed the advantage of the selected eremomycin amide derivative in particular, as well as the chosen direction in general.

## 1. Introduction

The problem of antibiotic-resistant spread has been acute in recent years [1]. Gram-positive bacteria pose as much of a threat as Gram-negative bacteria, and the number of infections and deaths in the United States per year caused by methicillin-resistant *S. aureus* (MRSA), *Streptococcus pneumoniae*, and *Clostridium difficile* significantly exceeds those caused by the most dangerous resistant Gram-negative pathogens [2].

**Figure 1:**
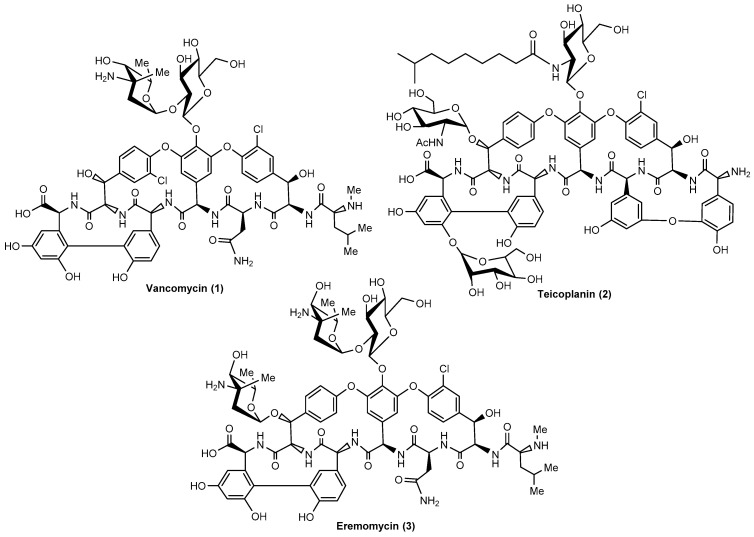
Structures of vancomycin, teicoplanin, and eremomycin.

The glycopeptide antibiotics (Figure 1) vancomycin (**1**) and teicoplanin (**2**) are used for the treatment of infections caused by MRSA, as well as penicillin-resistant strains of *S. pneumoniae* and *C. difficile* [3]. Although resistance to glycopeptide antibiotics has been developing for a long time, at present, it is a rather serious problem. Resistant strains, such as vancomycin-resistant enterococci (VRE), glycopeptide-resistant enterococci (GRE), and glycopeptide-resistant and glycopeptide-intermediate *S. aureus* (GISA), call into question the further use of vancomycin and even other glycopeptide antibiotics [4,5,6]. Thus, although both vancomycin and its new semi-synthetic analogs are currently limited in clinical practice, the development of new glycopeptide antibiotics (GPAs) is still relevant because of to the need to create reserve drugs that are active against resistant strains of bacteria.

A significant number of studies have been devoted to the effect of a hydrophobic fragment on the activity of semi-synthetic glycopeptides [7,8,9]. It has been shown that the introduction of a hydrophobic moiety in a glycopeptide molecule possibly increases activity against glycopeptide-resistant strains, e.g., in the case of oritavancin [10]. Presumably, the activity against resistant strains is realized through co-binding to the D-Ala-D-Lac fragment—dimerization and anchoring on the membrane compensates for the low affinity of the binding pocket for the peptidoglycan fragment [11]. Alternative conceptions suggest the implementation of a different mechanism of action that does not depend on binding to the peptidoglycan residue [12,13,14]. The main drawback of all GPA hydrophobic derivatives is their low solubility in water; however, this drawback is compensated for by the introduction of an additional hydrophilic fragment [15,16]. The effect of various types of substituents and their positions on the activity of a semisynthetic glycopeptide is of considerable interest.

Eremomycin (**3**) is a glycopeptide antibiotic with a structure similar to vancomycin [17], except for its carbohydrate components, and it is more active than vancomycin against most strains of Gram-positive bacteria in vitro and in vivo [18]. Furthermore, studies carried out for the 2-adamantylamides of eremomycin and vancomycin have shown that against the same strains, eremomycin amide is 4–16 times more active, especially in the case of GRE strains [9,19]. Moreover, for some semisynthetic eremomycin amides, a lower allergenicity has been noted compared with eremomycin [20,21]. All of these findings make eremomycin amide derivatives promising for the development of a new generation of glycopeptide antibiotics with an improved efficiency and reduced adverse effects.

Thus, our main goal was to assess the antimicrobial potential and study the structure–activity relationship of new eremomycin aminoalkylamides, where various substituents containing hydrophobic fragments are linked to the eremomycin amide via a hydrophilic linker. We previously studied the effect of the introduction of a basic diamine fragment on the activity of semi-synthetic amides of eremomycin [22]. Our analysis showed that ethylenediamine is the most promising linker. Therefore, we synthesized new eremomycin aminoalkylamides that were elongated with benzyl groups containing various substituents, and performed antibacterial activity comparisons of the new compounds on a wide panel of Gram-positive strains, sensitive and resistant to «gold-standard» vancomycin. Based on the data obtained, the structure–activity relationships were investigated, and a lead compound was selected for in-depth testing. Research carried out using an in vivo model of *staphylococcus* sepsis, toxicity studies, and the estimated therapeutic index also showed the advantage of the selected semisynthetic eremomycin derivative in particular, as well as the chosen direction in general.

## 2. Results

**Scheme 1:**
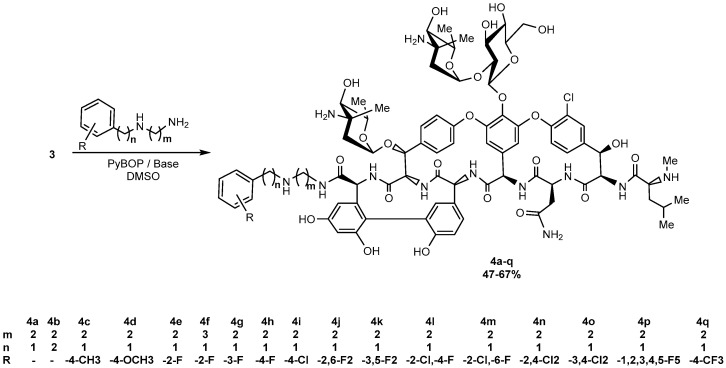
Synthesis of new eremomycin amides **4a**–**q**.

A series of eremomycin amides **4a**–**q** was prepared by the condensation of eremomycin with the corresponding amines according to the procedure described below, and were characterized by HRMS (high-resolution mass spectrometry) and NMR-spectroscopy with the purity confirmed by HPLC (high-performance liquid chromatography) and an elemental analysis (for **4e**). The derivatives were obtained in a good yield of 47–67%, and their general structure and synthesis scheme are shown in Scheme 1. The reaction was carried out under the conditions earlier determined as optimal for the amidation of eremomycin: 1:9:13:1.7 (eremomycin sulfate: dihydrochloride of the corresponding diamine: DIEA: PyBOP) [22]. The starting diamine dihydrochlorides were synthesized according to a known method [23]. Moreover, under these conditions, the acylation proceeded with a good chemoselectivity for the primary amino group of *N*-substituted diamines. The purification conditions for the new derivatives were optimized. Thus, the purification of the products was carried out by reversed-phase chromatography on an automatic Isolera system (Biotage, Uppsala, Sweden) on C18 Ultra cartridges, which gave final compounds with an appropriate purity (>95%).

Full ^13^C assignment is provided for the C-terminal tags for all F-containing compounds (Table S1), in accordance with the given structures (Scheme 1). In agreement with our recent work, [24] we conclude that all compounds in this study form asymmetric dimers in D_2_O solutions. This conclusion is corroborated by the existence of the aromatic 6e and 6e* aromatic CH groups around the typical 120 ppm ^13^C chemical shift; however, unusual ^1^H chemical shifts for these aromatic CH proton signals are clearly visible in all HSQC spectra (Figure S1). The unusual ^1^H chemical shift and signal doubling in the spectra can be explained by the formation of asymmetric dimers [25] (which includes orthogonal sigma–pi interactions at the back-to-back surface formed by appropriate aromatic rings). The asymmetry is caused by the overhanging disaccharide units that can slowly flip-flop their alignment in two orientations. Indeed, the ROESY spectra proved this slow exchange with opposite sign cross-peaks with respect to the real ROE peaks. The dimerization constants should have high values (strong, non-covalent dimers are formed), as the deuteration of the amide NH signals took days in several cases (data not shown). The presence of strong dimers allows for the determination of their 3D structure, which are deferred to another work. Here, we can provide a partial assignment for the compound **4e**, including some fingerprint signals (Table S2).

**Figure 2:**
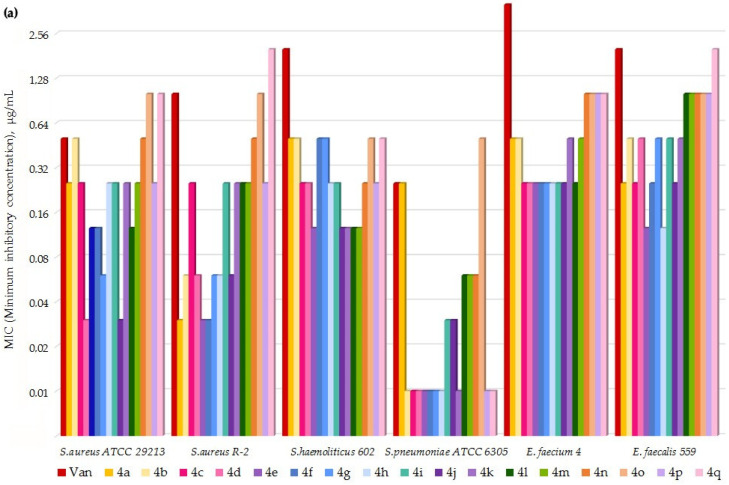

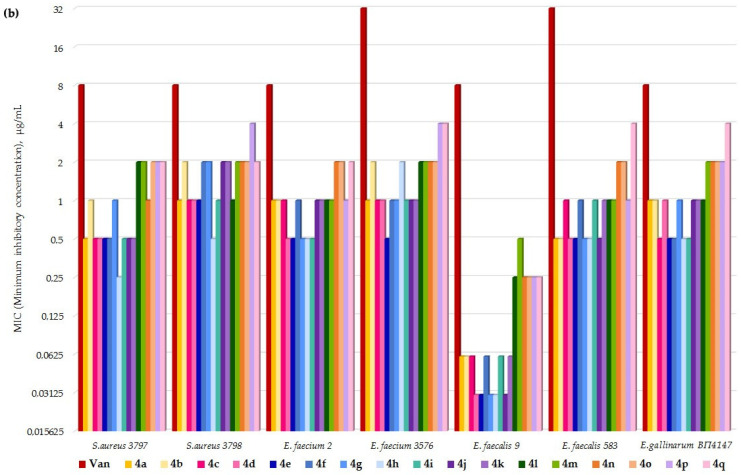
(**a**,**b**). Modal minimum inhibitory concentration (MIC) values of new amides **4a**–**q** and vancomycin, determined by the broth dilution micromethod for Gram-positive bacteria (**a**) susceptible and (**b**) with a reduced sensitivity to vancomycin.

For a comparative study of the antibacterial activity of new eremomycin derivatives **4a**–**q**, the MICs of new amides and vancomycin used in the clinic were tested on a control strain of *S. aureus* ATCC №29213, as well as a wide panel of Gram-positive bacteria (Table S3), the results of which are shown in Figure 2a,b and Tables S4 and S5. The screening results show that the new eremomycin carboxyl group modification can increase its activity against glycopeptide-resistant strains, while maintaining activity against susceptible strains.

For the selected lead-compound, eremomycin *N*-(2-((2-fluorobenzyl)amino)ethyl)amide (**4e**), a comparative study of the efficacy of the derivative and vancomycin was carried out on a mouse *staphylococcal* sepsis model for single intravenous (IV) administration. The research results are shown in Table 1.

An acute toxicity study was also performed for the lead-compound, eremomycin *N*-(2-((2-fluorobenzyl)amino)ethyl)amide (**4e**). The research results are shown in Table 2.

## 3. Discussion

The results of the activity studies against GPA-sensitive strains are shown in Figure 2a and Table S4. In general, it can be noted that derivatives **4c**–**k**, where benzyl is substituted with methyl, a methoxy group, chlorine, or one or two fluorine atoms, have a higher activity against susceptible strains than derivatives **4n**–**q** containing two chlorine atoms, five fluorine atoms, or a trifluoromethyl group. Derivatives **4l**,**m** containing both fluorine and chlorine atoms in the benzene ring are more active than dichloro derivatives **4n**,**o**, but less active than difluoro derivatives **4j**,**k**. The activities of all new derivatives against GPA-resistant strains are significantly higher than that of vancomycin (Figure 2b and Table S5). Comparing the activities of the new derivatives, it can be noted that the potency against resistant strains of enterococci followed the same pattern as the activity against sensitive strains of Gram-positive bacteria—derivatives containing fluorine as a substituent in benzyl showed a slightly higher activity than chlorine-containing congeners.

Thus, 17 new eremomycin derivatives showed a similar but not equal activity against susceptible and resistant strains of Gram-positive bacteria. Obviously, the activity of the derivative is related to the type and number of substituents on the aromatic ring in the side chain. Among the presented derivatives, *N*-(2-((2-fluorobenzyl)amino)ethyl)amide of eremomycin (**4e**) showed the best summary activity; however, the activities of the derivatives containing fluorine in the meta- and para-position differed insignificantly, as well as the activities of amides containing two fluorine atoms in different positions of **4j**,**k**, while *N*-(2-((2,3,4,5,6-pentafluorobenzyl)amino)ethyl)amide of eremomycin (**4p**) was noticeably less active against both sensitive and resistant strains. The potency of eremomycin *N*-(2-(4-chlorobenzyl)amino)ethyl)amide (**4i**) was comparable to fluorine containing analog **4h**; however, derivatives containing two chlorine atoms in the benzene ring (**4n**,**o**) already had a significantly lower activity in comparison with derivatives **4j**,**k**. The activity of derivatives **4l**,**m**, containing both fluorine and chlorine atoms can be called intermediate.

In general, the activities of derivatives with mono-halogen-containing substituents were higher those that of alkyl-containing ones. Comparing the activities of the amides containing methyl (**4c**), the methoxy group (**4d**), and trifluoromethyl (**4q**) in the aromatic benzyl ring, showed that if the activities of compounds **4c**,**d** were comparable, then the activity of **4q** was noticeably lower.

As we discovered earlier, eremomycin *N*-(2-aminoethyl)amide was more active (in 1–2 dilutions) than its homolog (eremomycin *N*-(2-aminopropyl)amide) [22]. In accordance with this data, analogue **4f** with the ortho-fluorobenzyl group attached through a propyl linker was inferior against GPA-resistant strains in comparison with the congener **4e** with an ethylene spacer.

Additionally, to make sure the results were clear, we obtained homologues **4a** and **4b** with an altered alkylene group attached the benzene ring to the ethylenediamine residue. Although both derivatives demonstrated a good activity, benzyl derivative **4a** was. in the majority cases, more active in one dilution than phenethyl derivative **4b**.

The best compounds of the series (**4d**–**h**) exhibited a similar activity in the range of one to two dilutions against both GPA-sensitive and -resistant strains. However, none one showed a maximum activity against for all strains. Therefore, the lead-compound was selected based on the general level of antibacterial activity against the used panel of pathogens. From the above series, the derivative was eremomycin *N*-(2-((2-fluorobenzyl)amino)ethyl)amide (**4e**), which was selected to further evaluate in vivo efficacy and toxicity.

A comparative study of the antibacterial efficacy that was carried out for the lead compound **4e**, «gold standard» vancomycin, and paternal eremomycin on a mouse *staphylococcal* sepsis model confirmed the prospects of the selected compound. The results of this study showed (Table 1) that the survival rate of the mice infected with *S. aureus* increased after single-dose IV injections of **4e**, vancomycin, or eremomycin in a dose-dependent manner. Based on the experimental data on the survival of the mice, the values of the effective doses (ED_50_) of the tested drugs were calculated (Table 1). The ED_50_ values demonstrated that the selected amide **4e** was 7.5 times more effective than the reference vancomycin and 3.3 times more active than the original eremomycin.

For the characterization of the therapeutic potential of the lead compound **4e**, an acute toxicity test for an IV single-dose administration was also carried out (Table 2). After the IV injection of the highest dose (200 mg/kg) of **4e**, the animals died immediately (“on the needle”). The drug administration in doses of 130–190 mg/kg led to the death of some animals from 2 to 15 min after injections as a result of cardiac and respiratory arrest against the background of neurotoxicity. No delayed death of animals was observed. The quantitative parameters of acute toxicity (LD_50_ and MTD (LD_10_) values) found in this study for **4e** are presented in Table 2. We calculated the LD_50_ as 175 mg/kg. In our previous investigation, the LD_50_ value for vancomycin on mice after IV administration was calculated as 525 mg/kg [21]. This data correlated well with the results reported by US Pharmacopeia [26,27]. Based on the received data, the therapeutic indexes (for laboratory animals testing) are as follows:

- Eremomycin *N*-(2-((2-fluorobenzyl)amino)ethyl)amide (**4e**) TI = 175/0.55 = 318;
- Vancomycin TI = 525/4.1 = 128.

Thus, according to the initial assessment, eremomycin *N*-(2-((2-fluorobenzyl)amino)ethyl)amide (**4e**) has a high therapeutic index, which exceeds the TI for vancomycin by 2.7 times.

## 4. Materials and Methods

The reagents and solvents were from Sigma-Aldrich. Eremomycin sulfate (95% purity), produced by the *Amicalatopsis Orientalys* strain, was obtained at the pilot plant of the FSBI Gause Institute of New Antibiotics (Moscow, Russian Federation). The purification of substances by reverse phase chromatography was carried out on an Isolera™ Prime (Biotage) automatic system, SNAP C18 Ultra cartridges (Boitage, 12 g), maximum working pressure 10 bar, UV detection (254–280 nm). An analysis of the substance’s purity by HPLC was carried out on a Shimadzu LC-20 AD chromatograph, on a Kromasil-100-5-μm C-18 column (4.6 × 250 mm), LW = 260 nm, eluent: A—H_3_PO_4_, B—MeCN (gradient B). High-resolution ESI mass spectra were recorded on a micrOTOF-Q II spectrometer (Bruker Daltonics, Billerica, MA, USA). The measurement accuracy was 0.25–0.38 in the mass range 118.086255–2721.894829. Positively charged ions were detected under the following conditions: capillary voltage 4 kV, atomizing nitrogen 40 kPa, drying gas flowrate 4 L/min, and source temperature 180 °C.

All of the NMR spectra were recorded in D_2_O at 288 K using a Bruker NEO/Avance III 700 MHz spectrometer (Bruker, Billerica, MA, USA) equipped with a high sensitivity, liquid nitrogen cooled (prodigy) triple-resonance probehead. Typical 90° pulses were 9 and 32 μs for ^1^H and ^13^C, and relaxation delays were typically 1.5 s. ^1^H-^13^C Heteronuclear Single Quantum Correlation (HSQC) spectra were recorded using «hsqcedetgpsp.3» pulse program using eight scans for each of the 640 increments in indirect dimension. The 2D spectra were processed using Topspin 3.1 software using Gaussian window function (Lb -5, GB 0.05) in F2 and cosine-square (QSINE, SSB = 2) in F1 dimension. To aid the ^1^H/^13^C assignments. heteronuclear multiple bond correlation experiments, HMBC (pulse program: “hmbcgplpndqf”, 50 ms evolution time), Heteronuclear Single Quantum Coherence-Total Correlated Spectroscopy (HSQC-TOCSY; pulse program: “hsqcdietgpsi”, mixing time 70 ms), homonuclear correlated spectroscopy (COSY) with water presaturation (pulse program: “cosygpprqf”), and Rotating Frame Overhauser Effect Spectroscopy (ROESY; pulse program: roesyadjsphpr, mixing time 100 ms). The digital resolution of the processed spectra was typically 2–3 Hz.

### 4.1. Synthesis, General Procedure

All of the derivatives were obtained according to the following general procedure.

To anhydrous dissolved in DMSO (12.5 mL), eremomycin sulfate (0.29 mmol) and DIPEA (3.77 mmol), dihydrochloride of substituted diamine (2.61 mmol), and PyBOP (0.49 mmol) were added, and the solution was stirred for 1 h. Isopropanol (8.5 mL), acetone (25 mL), and diethyl ether (15 mL) were added to the stirred solution and then filtered. The technical product was dissolved in 3–5 mL of water and purified by reversed-phase chromatography on the Isolera™ Prime (Biotage) automatic system (distilled water-acetonitrile). The fractions containing the product were combined, concentrated in vacuo to a volume of ~5 mL, after which the product was precipitated with acetone (50 mL) and dried in vacuum.

### 4.2. Compounds Characterization

- Eremomycin *N*-(2-(benzylamino)ethyl)amide (**4a**). Yield 47%. HPLC (column Kromasil-100-5-μm C-18 4.6 × 250 mm, LW = 260 nm, eluent: A—HCOONH_4_ (0.2%) pH = 4.5, B—MeCN; gradient B 10 → 35% (30 min): Rt = 8.8 min (96.9%). HRSM (ESI) calculated for C_82_H_102_ClN_12_O_25_ [M + H]^+^: 1689.6762; found 1689.6787.
- Eremomycin *N*-(2-phenethylamino)ethyl)amide (**4b**). Yield 48%. HPLC (column Kromasil-100-5-μm C-18 4.6 × 250 mm, LW = 260 nm, eluent: A—HCOONH_4_ (0.2%) pH = 4.5, B—MeCN; gradient B 10 → 35% (30 min): Rt = 12.7 min (95.4%). HRSM (ESI) calculated for C_83_H_104_ClN_12_O_25_ [M + H]^+^: 1703.6919; found 1703.6934.
- Eremomycin *N*-(2-((4-methylbenzyl)amino)ethyl)amide (**4c**). Yield 44%. HPLC (column Kromasil-100-5-μm C-18 4.6 × 250 mm, LW = 260 nm, eluent: A—HCOONH_4_ (0.2%) pH = 4.5, B—MeCN; gradient B 5 → 60% (30 min): Rt = 13.2 min (96.7%). HRSM (ESI) calculated for C_83_H_104_ClN_12_O_25_ [M + H]^+^: 1703.6919; found 1703.6905.
- Eremomycin *N*-(2-((4-methoxybenzyl)amino)ethyl)amide (**4d**). Yield 60%. HPLC (column Kromasil-100-5-μm C-18 4.6 × 250 mm, LW = 260 nm, eluent: A—HCOONH_4_ (0.2%) pH = 4.5, B—MeCN; gradient B 5 → 60% (30 min): Rt = 12.3 min (97.2%). HRSM (ESI) calculated for C_83_H_104_ClN_12_O_26_ [M + H]^+^: 1719.6868; found 1719.6882.
- Eremomycin *N*-(2-((2-fluorobenzyl)amino)ethyl)amide (**4e**). Yield 54%. HPLC (column Kromasil-100-5-μm C-18 4.6 × 250 mm, LW = 260 nm, eluent: A—HCOONH_4_ (0.6%) pH = 7.8, B—MeCN; gradient B 24–30% (30 min): Rt = 9.4 min (97.6%). HRSM (ESI) calculated for C_82_H_101_ClFN_12_O_25_ [M + H]^+^: 1707.6668; found 1707.6677. Found, %: C 50.19, H 5.96, N 8.57; calculated for C_82_H_100_ClFN_12_O_25*_4HCl_*_6H2O, %: C 50.34, H 5.80, N 8.59.
- Eremomycin *N*-(2-((2-fluorobenzyl)amino)propyl)amide (**4f**). Yield 58%. HPLC (column Kromasil-100-5-μm C-18 4.6 × 250 mm, LW = 260 nm, eluent: A—HCOONH_4_ (0.2%) pH = 4.5, B—MeCN; gradient B 10–35% (30 min): Rt = 8.8 min (98.1%). HRSM (ESI) calculated for C_83_H_103_ClFN_12_O_25_ [M + H]^+^: 1721.6824; found 1721.6798.
- Eremomycin *N*-(2-((3-fluorobenzyl)amino)ethyl)amide (**4g**). Yield 52%. HPLC (column Kromasil-100-5-μm C-18 4.6 × 250 mm, LW = 260 nm, eluent: A—HCOONH_4_ (0.2%) pH = 4.5, B—MeCN; gradient B 5 → 60% (30 min): Rt = 12.2 min (96.4%). HRSM (ESI) calculated for C_82_H_101_ClFN_12_O_25_ [M + H]^+^: 1707.6668; found 1707.6645.
- Eremomycin *N*-(2-((4-fluorobenzyl)amino)ethyl)amide (**4h**). Yield 57%. HPLC (column Kromasil-100-5-μm C-18 4.6 × 150 mm, LW = 270 nm, eluent: A—HCOONH_4_ (0.2%) pH = 6.5, B—MeCN; gradient B, 5–60% (30 min): Rt = 20.9 min (96.7%). HRMS (ESI) calculated for C_82_H_101_ClFN_12_O_25_ [M + H]^+^: 1707.6668; found 1707.6653.
- Eremomycin *N*-(2-((4-chlorobenzyl)amino)ethyl)amide (**4i**). Yield 50%. HPLC (column Kromasil-100-5-μm C-18 4.6 × 250 mm, LW = 260 nm, eluent: A—HCOONH_4_ (0.2%) pH = 4.5, B—MeCN; gradient B 5 → 60% (30 min): Rt = 14.2 min (95.6%). HRSM (ESI) calculated for C_82_H_101_Cl_2_N_12_O_25_ [M + H]^+^: 1723.6372; found 1723.6364.
- Eremomycin *N*-(2-((2,6-difluorobenzyl)amino)ethyl)amide (**4j**). Yield 48%. HPLC (column Kromasil-100-5-μm C-18 4.6 × 250 mm, LW = 260 nm, eluent: A—HCOONH_4_ (0.2%) pH = 4.5, B—MeCN; gradient B 5 → 60% (30 min): Rt = 14.0 min (95.4%). HRSM (ESI) calculated for C_82_H_100_ClF_2_N_12_O_25_ [M + H]^+^: 1725.6574; found 1725.6534.
- Eremomycin *N*-(2-((3,5-difluorobenzyl)amino)ethyl)amide (**4k**). Yield 49%. HPLC (column Kromasil-100-5-μm C-18 4.6 × 250 mm, LW = 260 nm, eluent: A—HCOONH_4_ (0.2%) pH = 4.5, B—MeCN; gradient B 10 → 35% (30 min): Rt = 11.9 min (95.3%). HRSM (ESI) calculated for C_82_H_100_ClF_2_N_12_O_25_ [M + H]^+^: 1725.6574; found 1725.6546.
- Eremomycin *N*-(2-((2-chloro-4-fluorobenzyl)amino)ethyl)amide (**4l**). Yield 45%. HPLC (column Kromasil-100-5-μm C-18 4.6 × 250 mm, LW = 260 nm, eluent: A—HCOONH_4_ (0.2%) pH = 4.5, B—MeCN; gradient B 10 → 60% (30 min): Rt = 10.4 min (97.3%). HRSM (ESI) calculated for C_82_H_100_Cl_2_FN_12_O_25_ [M + H]^+^: 1741.6278; found 1741.6260.
- Eremomycin *N*-(2-((2-chloro-6-fluorobenzyl)ethyl)amide (**4m**). Yield 46%. HPLC (column Kromasil-100-5-μm C-18 4.6 × 250 mm, LW = 260 nm, eluent: A—HCOONH_4_ (0.2%) pH = 4.5, B—MeCN; gradient B 10 → 35% (30 min): Rt = 10.7 min (96.8%). HRSM (ESI) calculated for C_82_H_100_Cl_2_FN_12_O_25_ [M + H]^+^: 1741.6278, found 1741.6253.
- Eremomycin *N*-(2-((2,4-dichlorobenzyl)amino)ethyl)amide (**4n**). Yield 61%. HPLC (column Kromasil-100-5-μm C-18 4.6 × 250 mm, LW = 260 nm, eluent: A—HCOONH_4_ (0.2%) pH = 4.5, B—MeCN; gradient B 5 → 60% (30 min): Rt = 15.2 min (97.1%). HRSM (ESI) calculated for C_82_H_100_Cl_3_N_12_O_25_ [M + H]^+^: 1757.5983; found 1757.5883.
- Eremomycin *N*-(2-((3,4-dichlorobenzyl)amino)ethyl)amide (**4o)**. Yield 48%. HPLC (column Kromasil-100-5-μm C-18 4.6 × 250 mm, LW = 260 nm, eluent: A—HCOONH_4_ (0.2%) pH = 4.5, B—MeCN; gradient B 5 → 60% (30 min): Rt = 16.1 min (95.7%). HRSM (ESI) calculated for C_82_H_100_Cl_3_N_12_O_25_ [M + H]^+^: 1757.5983; found 1757.6009.
- Eremomycin *N*-(2-((2,3,4,5,6-pentafluorobenzyl)amino)ethyl)amide (**4p**). Yield 63%. HPLC (column Kromasil-100-5-μm C-18 4.6 × 250 mm, LW = 260 nm, eluent: A—HCOONH_4_ (0.2%) pH = 4.5, B—MeCN; gradient B 10 → 35% (30 min): Rt = 16.7 min (96.6%). HRSM (ESI) calculated for C_82_H_97_ClF_5_N_12_O_25_ [M + H]^+^: 1779.6291; found 1779.6276.
- Eremomycin *N*-(2-((4-trifluoromethylbenzyl)amino)ethyl)amide (**4q**). Yield 52%. HPLC (column Kromasil-100-5-μm C-18 4.6 × 250 mm, LW = 260 nm, eluent: A—HCOONH_4_ (0.2%) pH = 4.5, B—MeCN; gradient B 10 → 60% (30 min): Rt = 13.3 min (95.2%). HRSM (ESI) calculated for C_83_H_101_ClF_3_N_12_O_25_ [M + H]^+^: 1757.6636; found 1757.6802.

### 4.3. MIC Values Determination

The antimicrobial activity of semisynthetic derivatives of eremomycin **4a**–**q** was studied in comparison with vancomycin on a wide panel of Gram-positive bacteria sensitive (*Staphylococcus aureus* ATCC 29213, *S. aureus* R-2, *S. haemoliticus* 602, *S. pneumoniae* ATCC 6305, *Enteroccus faecium* 4, and *E. faecalis* 559) and resistant to vancomycin (*S. aureus* 3797, *S.aureus* 3798, *E. faecium* 2, *E. faecium* 3576, *E. faecalis* 9, *E. faecalis* 583, and *E. gallinarum* BΠ 4147), information about the strains given in Table S3. The minimum microbial inhibitory concentration (MIC) for the test compounds was determined by the broth dilution micromethod according to CLSI guidelines [28], and vancomycin and a standard strain *S. aureus* ATCC 29213 used as controls (Figure 2 and Tables S4 and S5). The reproducibility of the results of three to five independent repetitions did not go beyond the one dilution, which is acceptable for this method.

### 4.4. In Vivo Efficiency Study

The animal study was performed in accordance with the European Convention for the Protection of Vertebrate Animals, Directives 86/609/EEC [29], the European Convention for Humane Methods for Animal Welfare and Maintenance [30], and the National Standard of the Russian Federation 33044–2014 “Good Laboratory Practice” [31], and was approved by the Ethics of Animal Experimentation of the Gause Institute of New Antibiotics.

A comparative study of the efficacy of eremomycin *N*-(2-((2-fluorobenzyl)amino)ethyl)amide (**4e**) and vancomycin was carried out in a mice *staphylococcal* sepsis model. Healthy female mice of the SHK colony weighing 20–22 g after two-week quarantine were randomized into groups (*n* = 10) and received *S. aureus* as the infectious agent (strain 10, clinical isolate, adapted for growth in vivo by five-fold passaging in mice). In the experiment, female mice of the SHK colony weighing 20–22 g were used. *S. aureus* (strain 10, clinical isolate), adapted for growth in vivo by five-fold passaging in mice, was used as the infectious agent. Initially, the lethal dose (LD_100_) of staphylococcus was determined for this mouse line using the intravenous infection route. The mice deaths were counted daily for 10 days. Thus, the lethal dose (LD_100_) was defined as 8 × 10^8^ CFU/mouse. Afterwards, the mice were seated in cages of 10 heads and were infected intravenously with *S. aureus* at a lethal dose, and the efficacy of the tested drugs was determined by the ED_50_ value (i.e., the dose at which 50% of the experimental animals survive). Then, 30 min after infection, the mice were injected intravenously with eremomycin *N*-(2-((2-fluorobenzyl)amino)ethyl)amide (**4e**) at single doses from 0.1 to 2.5 mg/kg. or vancomycin at doses from 2.5 to 7.5 mg/kg. As a control dose, a group of untreated animals infected with a lethal dose of *S. aureus* was present in the experiment. The determination of ED_50_ of the tested compounds was carried out in one experiment under a single control using the Behrens method [32]. The animals were observed for 14 days, and the deaths were counted daily.

### 4.5. Acute Toxicity

The F1 (CBAxC57Bl) mice [33] (18–20 g) were randomized into groups (*n* = 6) and received eremomycin *N*-(2-((2-fluorobenzyl)amino)ethyl)amide **4e** through single intravenous injections. The intact animals were used as the control. The substance was dissolved in a 5% glucose solution and was administrated in dosage ranges of 130–200 mg/kg into the tail veins of the mice. The concentration of **4e** in the injected solution was 5 mg/mL.

The acute toxicity was estimated by the mortality and survival time, as well as by the body weight gain/loss, food consumption, and clinical symptoms of intoxication, including behavioral reactions. The animals were observed for 30 days after the last death case, and then the surviving animals were euthanized and subjected to necropsy for examination of the internal abnormalities. The LD_50_ values and the maximum tolerated doses (MTD = LD_10_) were calculated using the method of Litchfield and Wilcoxon using the StatPlus Professional 3.8.0 software.

## 5. Conclusions

In the course of this work, we prepared and characterized 17 new eremomycin amides in which eremomycin was conjugated with an aromatic ring of substituted benzyl via diamine residue. All of the new amides of eremomycin were more active than vancomycin against both susceptible and GPA-resistant strains. In general, the best activity among the new derivatives was possessed by derivatives containing a fluorobenzyl group. For the selected lead compound **4e**, an in vivo efficacy study was carried out for IV single dose administration in a model of the mice *staphylococcal* sepsis. The studies showed that in vivo eremomycin *N*-(2-((2-fluorobenzyl)amino)ethyl)amide (**4e**) has an ED_50_ that is 7.5 times lower than the «gold standard» vancomycin. The results of the estimation of the acute toxicity of **4e** for an IV single dose demonstrated that the therapeutic index of the selected lead compound was 317, which is 2.7 times higher than that for vancomycin.

All of the obtained data support new prospects for further work in this direction. It is important and interesting to obtain a series of derivatives with different structures and substituent natures on the benzyl aromatic ring in order to study the full spectrum of structure–activity bonds. More in-depth studies of efficacy, toxicity, and pharmacokinetic propensities of the promising lead-compound **4e** are under way.

## Figures and Tables

**Table 1:** Efficacy (ED_50_, mg/kg) of eremomycin *N*-(2-((2-fluorobenzyl)amino)ethyl)amide (**4e**), vancomycin (**1**), and eremomycin (**3**) on a mouse model of staphylococcal sepsis.

Compound.	Dose, mg/kg	Survival, %	ED_50_, mg/kg
Eremomycin *N*-2-((2-fluorobenzyl)amino)ethyl) amide (**4e**)	0.1	20	0.55
	0.25	30	
	0.5	50	
	1	60	
	1.5	80	
	2.5	100	
Vancomycin (**1**)	2.5	20	4.1
	3.5	40	
	4.5	50	
	5.5	80	
	6.5	90	
	7.5	100	
Eremomycin (**3**)	0.5	20	1.8
	1.5	40	
	2.5	80	
	3.5	80	
	4.5	100	
	5.5	100	
Control dose *S. aureus*	-	0	

**Table 2:** Acute toxicity of LD_50_ and MTD (LD_10_) of *N*-(2((2-fluorobenzyl)amino)ethyl)amide (**4e**) in mice.

Parameter	4e
LD_50_ (mg/kg)	175.5 (161.7 ÷ 189.8) *
MTD (LD_10_) (mg/kg)	144.7

Note: * Data shown as mean LD_50_ (significant deviation of LD_50_ for *p* ≤ 0.05). Abbreviations: LD_10_, 10% lethal dose; LD_50_, 50% lethal dose; MTD, maximum tolerated dose.

## Data Availability

More data from this work are available in the Supplementary Material.

## Supplementary Materials

The following are available online at https://www.mdpi.com/article/10.3390/ph14040379/s1. Figure S1: Atom’s numeration for ^13^C nuclear magnetic resonance spectra of eremomycin *N*-(2-((2-fluorobenzyl)amino)ethyl)amide (**4e**). Figures S2–S19: ^1^H-^13^C correlation, 288K (D2O, 700 MHz ^1^H-NMR Spectrometer) and 13C-NMR (288K, D2O, 700 MHz) for F-containing compounds. Table S1: C-terminal tags in eremomycin derivatives, ^13^C assignments (^1^H shifts, in case of ^13^C overlap). Multiplicity of ^13^C signals due to ^19^F spin-spin couplings are given as d (doublet) and t (triplet). Table S2: Partial ^1^H/^13^C NMR signal assignment of compound **4e** including several fingerprint assignments. Table S3: Characterization of the strains used in the study. Table S4: Antibacterial activity of vancomycin and derivatives **4a**–**q** against sensitive Gram-positive bacteria. Table S5: Antibacterial activity of vancomycin and derivatives **4a**–**q** against resistant Gram-positive bacteria.

## References

[1] Asokan G.V., Ramadhan T., Ahmed E., Sanad H. (2019). WHO Global Priority Pathogens List: A Bibliometric Analysis of Medline-PubMed for Knowledge Mobilization to Infection Prevention and Control Practices in Bahrain. Oman Med. J..

[2] Antibiotic Resistance Threats in the United States, 2019 (2019 AR Threats Report). https://www.cdc.gov/drugresistance/pdf/threats-report/2019-ar-threats-report-508.pdf.

[3] Zeng D., Debabov D., Hartsell T.L., Cano R.J., Adams S., Schuyler J.A., McMillan R., Pace J.L. (2016). Approved Glycopeptide Antibacterial Drugs: Mechanism of Action and Resistance. Cold Spring Harb. Perspect. Med..

[4] Vehreschild M.J.G.T., Haverkamp M., Biehl L.M., Lemmen S., Fätkenheuer G. (2019). Vancomycin-resistant enterococci (VRE): A reason to isolate?. Infection.

[5] Dancer S.J. (2003). Glycopeptide resistance in Staphylococcus aureus. J. Antimicrob. Chemother..

[6] Hubert S.K., Mohammed J.M., Fridkin S.K., Gaynes R.P., McGowan J.E., Tenover F.C. (1999). Glycopeptide-Intermediate Staphylococcus aureus: Evaluation of a Novel Screening Method and Results of a Survey of Selected U.S. Hospitals. J. Clin. Microbiol..

[7] Leadbetter M.R., Adams S.M., Bazzini B., Fatheree P.R., Karr D.E., Krause K.M., Lam B.M.T., Linsell M.S., Nodwell M.B., Pace J.L. (2004). Hydrophobic vancomycin derivatives with improved ADME properties: Discovery of telavancin (TD-6424). J. Antibiot..

[8] Zhanel G.G., Calic D., Schweizer F., Zelenitsky S., Adam H., Lagacé-Wiens P.R.S., Rubinstein E., Gin A.S., Hoban D.J., Karlowsky J.A. (2010). New Lipoglycopeptides. Drugs.

[9] Moiseenko E.I., Grammatikova N.E., Shchekotikhin A.E. (2019). Eremomycin Picolylamides and Their Cationic Lipoglycopeptides: Synthesis and Antimicrobial Properties. Macroheterocycles.

[10] Allen N.E., Nicas T.I. (2003). Mechanism of action of oritavancin and related glycopeptide antibiotics. FEMS Microbiol. Rev..

[11] Allen N.E., Letourneau D.L., Hobbs J.N. (1997). The Role of Hydrophobic Side Chains as Determinants of Antibacterial Activity of Semisynthetic Glycopeptide Antibiotics. J. Antibiot..

[12] Ge M., Chen Z., Russell H., Onishi H.R., Kohler J., Silver L.L., Kerns R., Fukuzawa S., Thompson C., Kahne D. (1999). Vancomycin Derivatives That Inhibit Peptidoglycan Biosynthesis Without Binding D-Ala-D-Ala. Science.

[13] Kerns R., Dong S.D., Fukuzawa S., Carbeck J., Kohler J., Silver L.L., Kahne D. (2000). The Role of Hydrophobic Substituents in the Biological Activityof Glycopeptide Antibiotics. J. Am. Chem. Soc..

[14] Printsevskaya S.S., Pavlov A.Y., Olsufyeva E.N., Mirchink E.P., Isakova E.B., Reznikova M.I., Goldman R.C., Branstrom A.A., Baizman E.R., Longley C.B. (2002). Synthesis and Mode of Action of Hydrophobic Derivatives of the Glycopeptide Antibiotic Eremomycin and Des-(N-methyl-d-leucyl)eremomycin against Glycopeptide-Sensitive and -Resistant Bacteria. J. Med. Chem..

[15] Pavlov A.Y., Preobrazhenskaya M.N., Malabarba A., Ciabatti R., Colombo L. (1998). Mono and Double Modified Teicoplanin Aglycon Derivatives on the Amino Acid No. 7; Structure-activity Relationship. J. Antibiot..

[16] Yasukata T., Shindo H., Yoshida O., Sumino Y., Munekage T., Narukawa Y., Nishitani Y. (2002). An efficient and practical method for solid-phase synthesis of tripeptide-bearing glycopeptide antibiotics: Combinatorial parallel synthesis of carboxamide derivatives of chloroorienticin B. Bioorganic Med. Chem. Lett..

[17] Gauze G.F., Brazhnikova M.G., Laĭko A.V., Sveshnikova M.A., Preobrazhenskaia T.P. (1987). Eremomycin—A new antibiotic from the cyclic glycopeptide group. Antibiot. I Meditsinskaia Biotekhnologiia Antibiot. Med. Biotechnol..

[18] Filippos’Iants S.T., Malkova I.V., Gol’Dberg L.E. (1989). Glycopeptide antibiotics: Eremomycin, vancomycin, and teicoplanin. Comparison of several parameters of pharmacokinetics and antimicrobial activity. Antibiot. I Khimioterapiia Antibiot. Chemoterapy.

[19] Maples K.R., Wheeler C., Ip E., Plattner J.J., Chu D., Zhang Y.-K., Preobrazhenskaya M.N., Printsevskaya S.S., Solovieva S.E., Olsufyeva E.N. (2007). Novel Semisynthetic Derivative of Antibiotic Eremomycin Active against Drug-Resistant Gram-Positive Pathogens IncludingBacillusanthracis. J. Med. Chem..

[20] Pavlov A.Y., Miroshnikova O.V., Printsevskaya S.S., Olsufyeva E.N., Preobrazhenskaya M.N., Goldman R.C., Branstrom A.A., Baizman E.R., Longley C.B. (2001). Synthesis of Hydrophobic N′-Mono and N′,N″-Double Alkylated Eremomycins Inhibiting the Transglycosylation Stage of Bacterial Cell Wall Biosynthesis. J. Antibiot..

[21] Olsufyeva E.N., Shchekotikhin A.E., Bychkova E.N., Pereverzeva E.R., Treshalin I.D., Mirchink E.P., Isakova E.B., Chernobrovkin M.G., Kozlov R.S., Dekhnich A.V. (2018). Eremomycin pyrrolidide: A novel semisynthetic glycopeptide with improved chemotherapeutic properties. Drug Des. Dev. Ther..

[22] Moiseenko E.I., Grammatikova N.E., Shchekotikhin A.E. (2020). Synthesis and Antibacterial Activity of Aminoalkylamides of Eremomycin. Macroheterocycles.

[23] Wang K., Qian X., Cui J. (2009). One step from nitro to oxime: A convenient preparation of unsaturated oximes by the reduction of the corresponding vinylnitro compounds. Tetrahedron.

[24] Izsépi L., Erdei R., Tevyashova A., Grammatikova N., Shchekotikhin A., Herczegh P., Batta G. (2021). Bacterial Cell Wall Analogue Peptides Control the Oligomeric States and Activity of the Glycopeptide Antibiotic Eremomycin: Solution NMR and Antimicrobial Studies. Pharmaceuticals.

[25] Groves P., Searle M.S., Mackay J.P., Williams D.H. (1994). The structure of an asymmetric dimer relevant to the mode of action of the glycopeptide antibiotics. Structure.

[26] Product Monograph–Vancomycin Hydrochloride for Injection, USP, 2017:21. https://www.fresenius-kabi.com/en-ca/documents/Vanco-for-Inj.-PM-ENG-v7.0-011218.pdf.

[27] Vancomycin Hydrochloride for Injection, USP, 2019:02. https://www.athenexpharma.com/wp-content/uploads/materials/SDS/Vancomycin_SDS.pdf.

[28] (2020). Clinical and Laboratory Standarts Institute M100 Performance Standards for Antimicrobial Susceptibility Testing. https://clsi.org/standards/products/microbiology/documents/m100/.

[29] Council of Europe European Convention for the Protection of Vertebrate Animals Used for Experimental and Other Purposes. https://rm.coe.int/168007a67b.

[30] Directive 2010/63/EU on the Protection of Animals Used for Scientific Purposes EN. https://eur-lex.europa.eu/LexUriServ/LexUriServ.do?uri=OJ:L:2010:276:0033:0079:EN:PDF.

[31] National State Standard GOST 33044-2014 the Russian Federation Standard “The Principles of Good Laboratory Practice” (Approved and Put into Effect by the Order of the Federal Agency for Technical Regulation and Metrology of October 20, 2014), No 1700. https://docs.cntd.ru/document/1200115791.

[32] Behrens B. (1929). Zur Auswertung der Digitalisblätter im Forschversuch. Arch. Exper. Path. Pharm..

[33] Binder M.D., Hirokawa N., Windhorst U. (2008). (CBAxC57BL/6)F1. Encyclopedia of Neuroscience.

