# Supplementary material for: Aminoalkylamides of Eremomycin Exhibit an Improved Antibacterial Activity
Source: Pharmaceuticals (Basel). 2021 Apr 19;14(4):379. doi: 10.3390/ph14040379 (PMC8072890; doi:10.3390/ph14040379)
Supplement: Supplementary file 1 [file pharmaceuticals-14-00379-s001.zip › pharmaceuticals-1176561 supplementary.pdf]

Aminoalkylamides of Eremomycin Exhibit Improved Antibacterial Activity

Elena I. Moiseenko[1], Réka Erdei[2], Natalia E. Grammatikova[1], Elena P. Mirchink[1], Elena B. Isakova[1], Eleonora R. Pereverzeva[1], Gyula Batta[2], Andrey E. Shchekotikhin[1*]

[1] Gause Institute of New Antibiotics, Moscow,119021, Russian Federation;

[2] Department of Organic Chemistry, University of Debrecen, H-4032 Debrecen, Egyetem tér 1, Hungary;

* Correspondence: shchekotikhin@gause-inst.ru

Supplementary Material

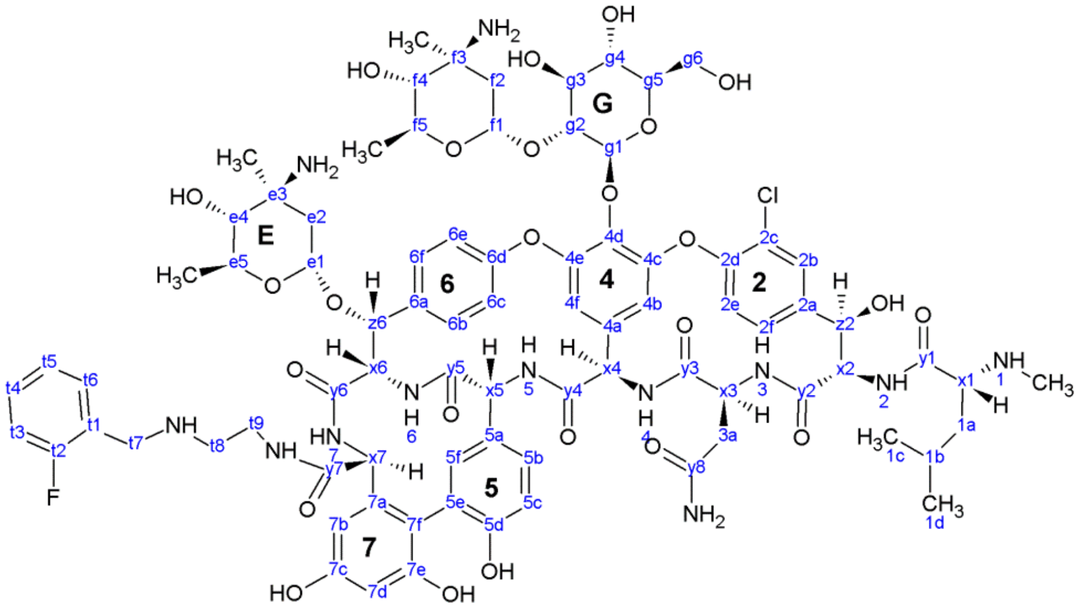

Figure S1. Atom's numeration for 13C nuclear magnetic resonance spectra of eremomycin N-(2-((2-fluorobenzyl)amino)ethyl)amide (**4e**).

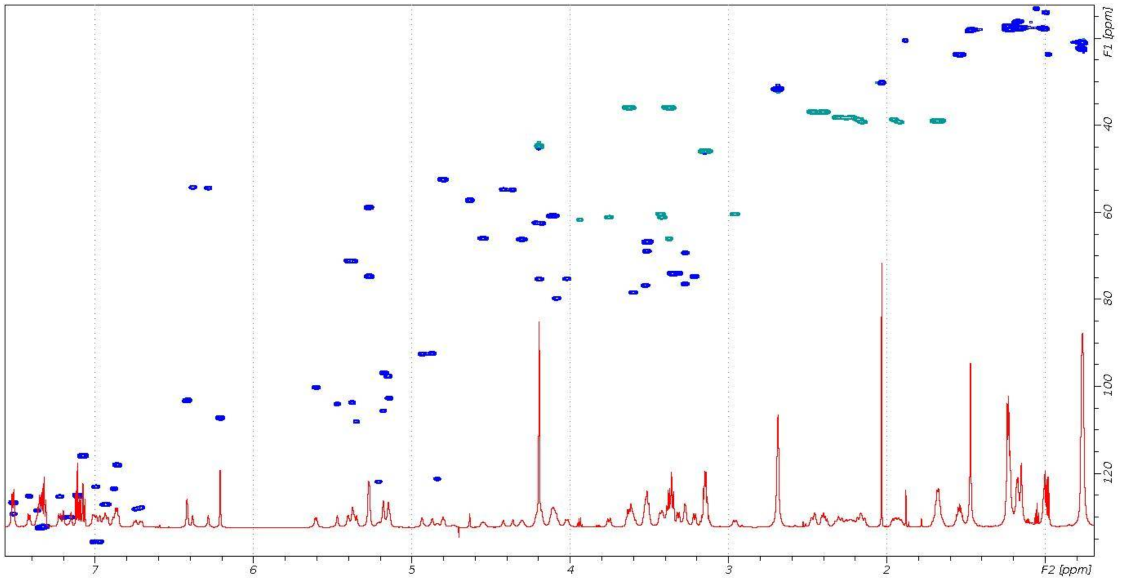

Figure S2. 4e, ^{1}H-^{13}C correlation, 288K, D2O, 700 MHz ^{1}H-NMR Spectrometer.

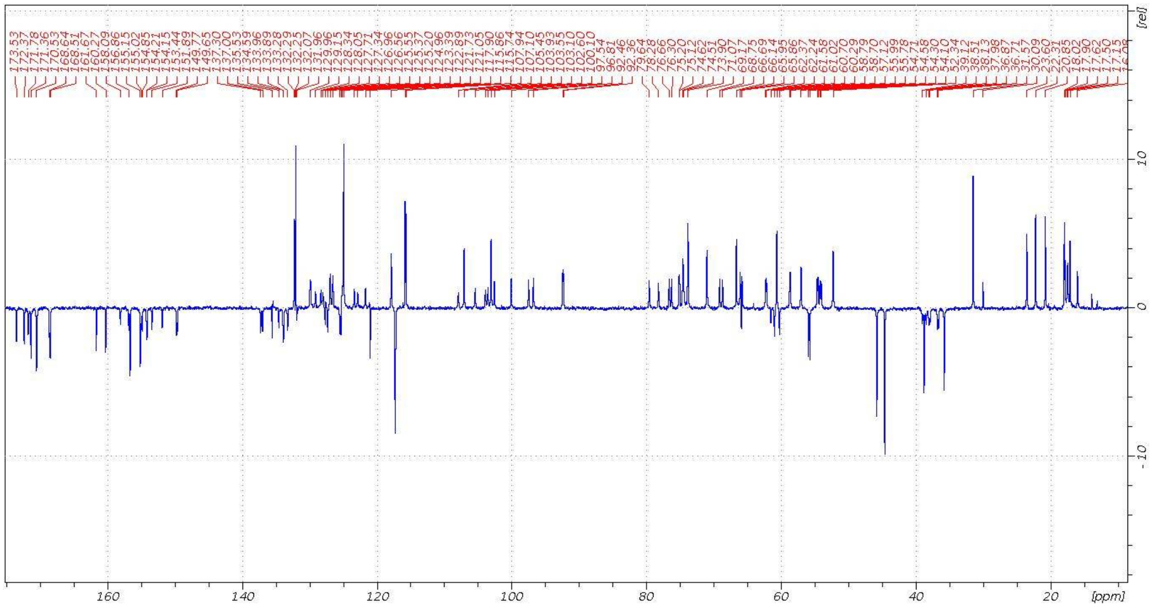

Figure S3. 4e, ^{13}C- NMR, 288K, D2O, 700 MHz.

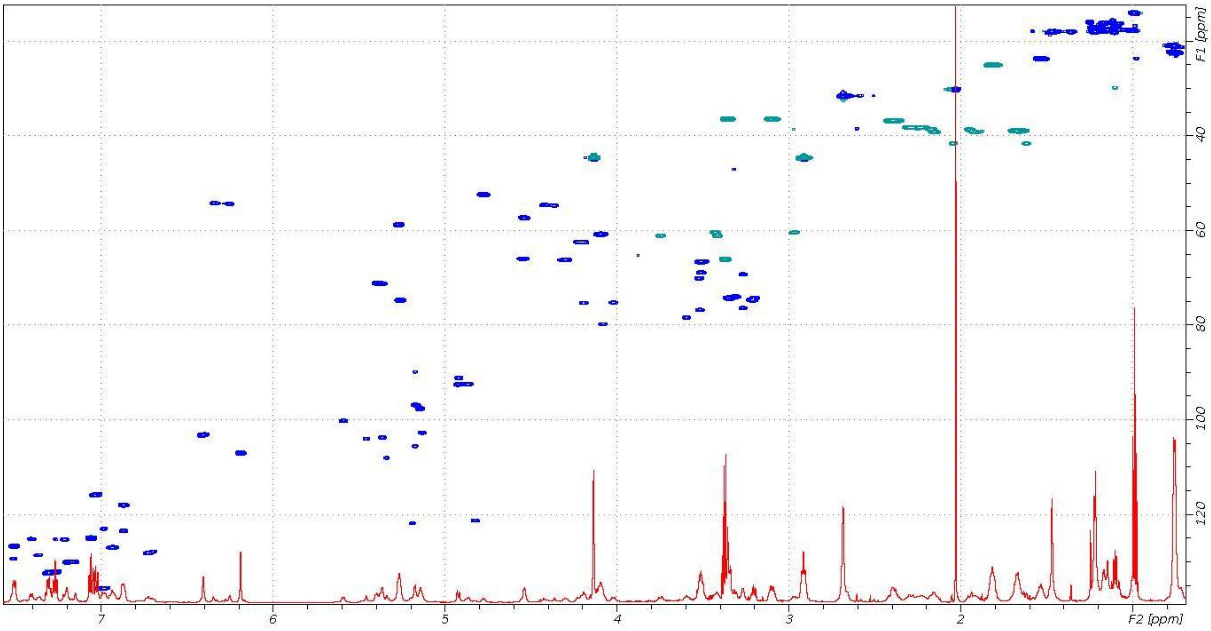

Figure S4. 4f, ^{1}H-^{13}C correlation, 288K, D2O, 700 MHz ^{1}H-NMR Spectrometer.

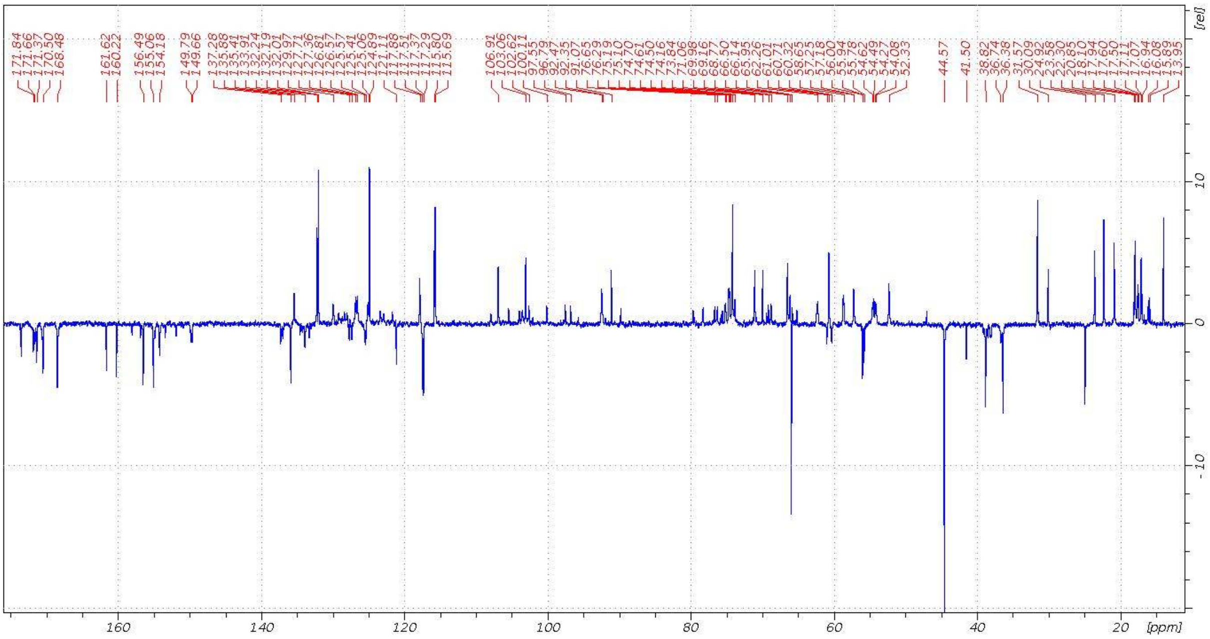

Figure S5. 4f, ^{13}C- NMR, 288K, D2O, 700 MHz.

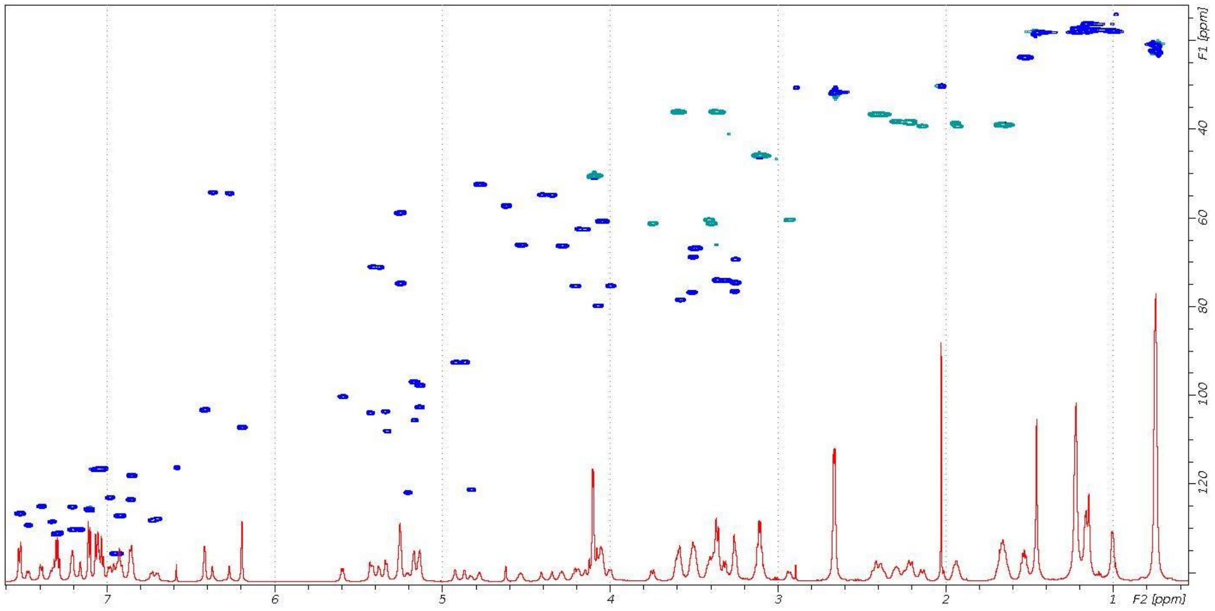

Figure S6. 4g, ^{1}H-^{13}C correlation, 288K, D2O, 700 MHz ^{1}H-NMR Spectrometer.

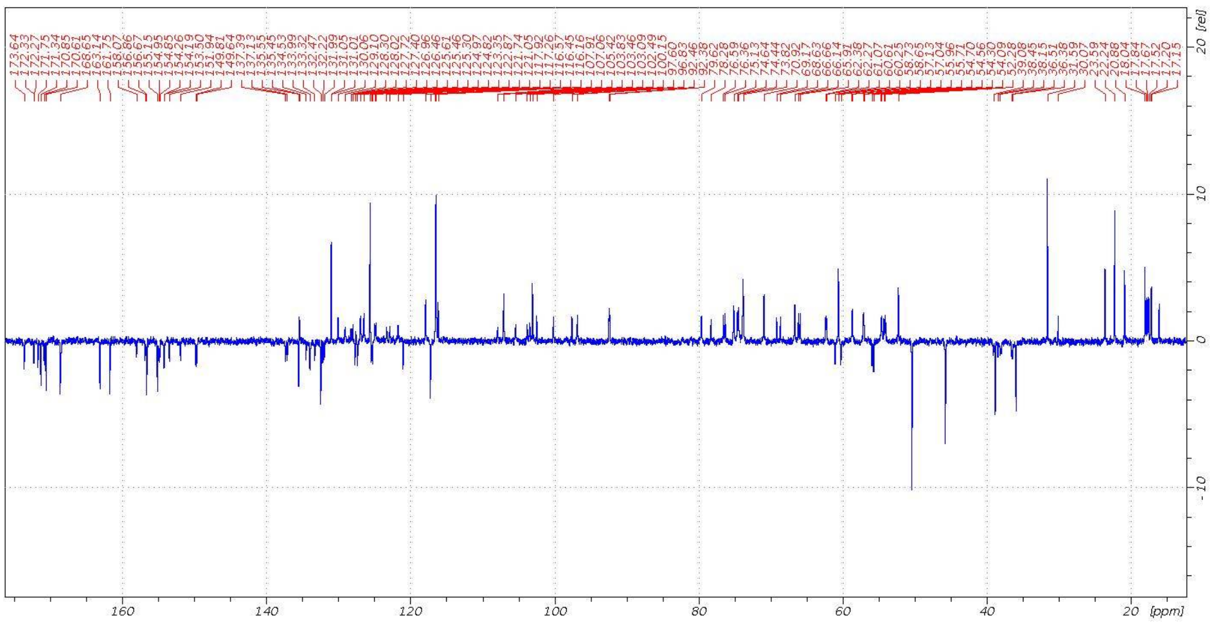

Figure S7. 4g, ^{13}C- NMR, 288K, D2O, 700 MHz.

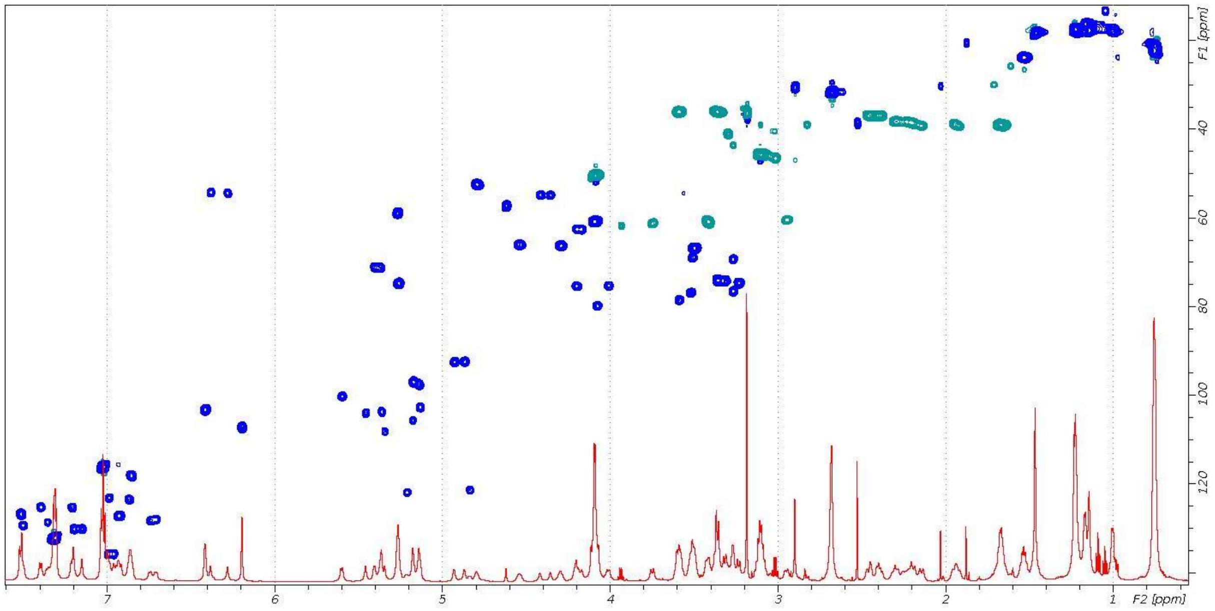

Figure S8. 4h, ^{1}H-^{13}C correlation, 288K, D2O, 700 MHz ^{1}H-NMR Spectrometer.

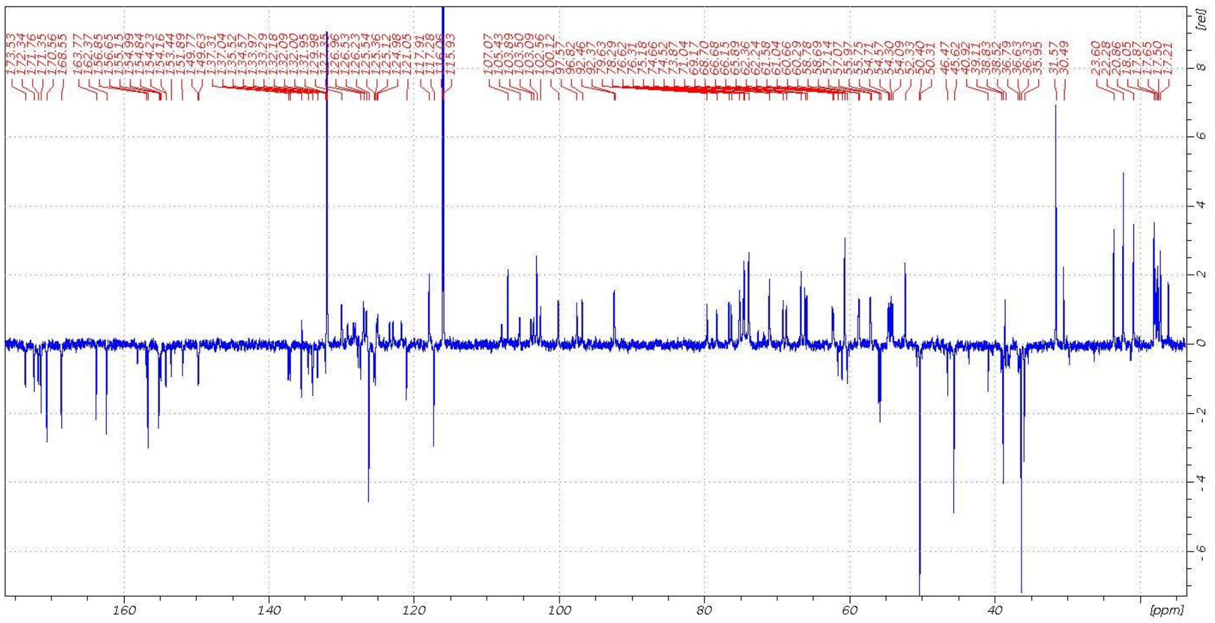

Figure S9. 4h, ^{13}C- NMR, 288K, D2O, 700 MHz.

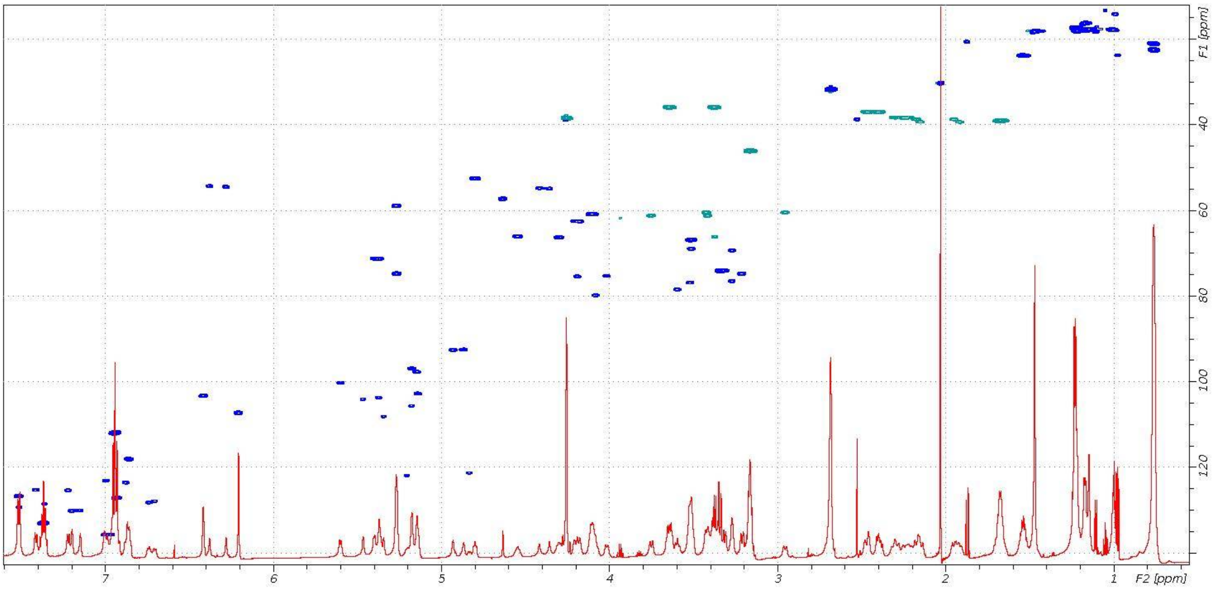

Figure S10. 4j, ^{1}H-^{13}C correlation, 288K, D2O, 700 MHz ^{1}H-NMR Spectrometer.

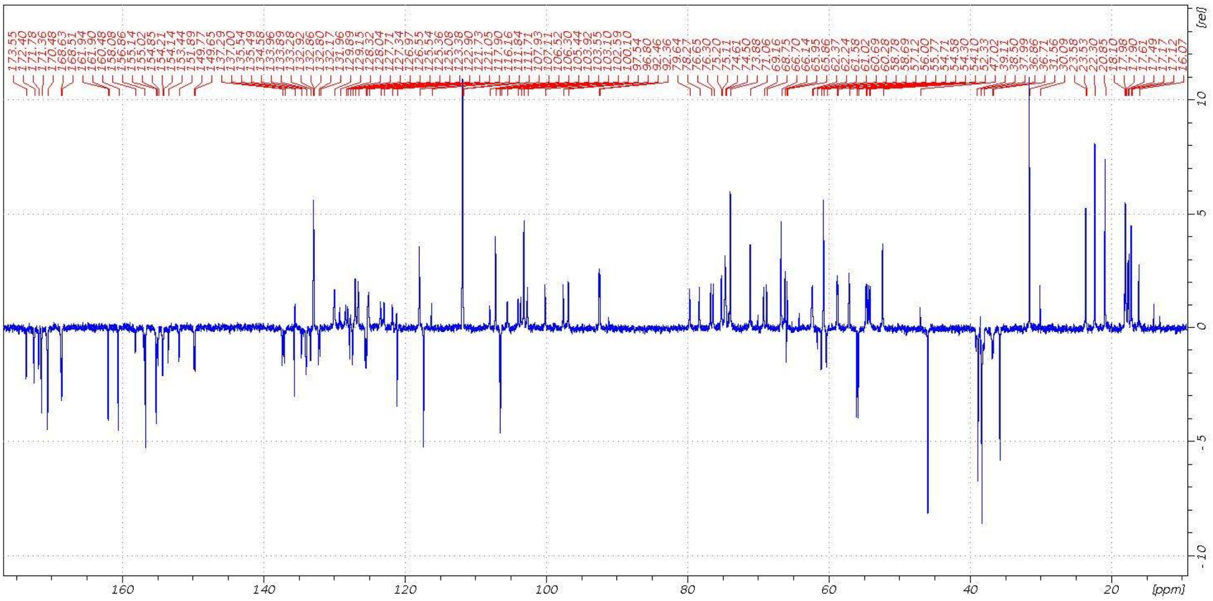

Figure S11. 4j, ^{13}C- NMR, 288K, D2O, 700 MHz.

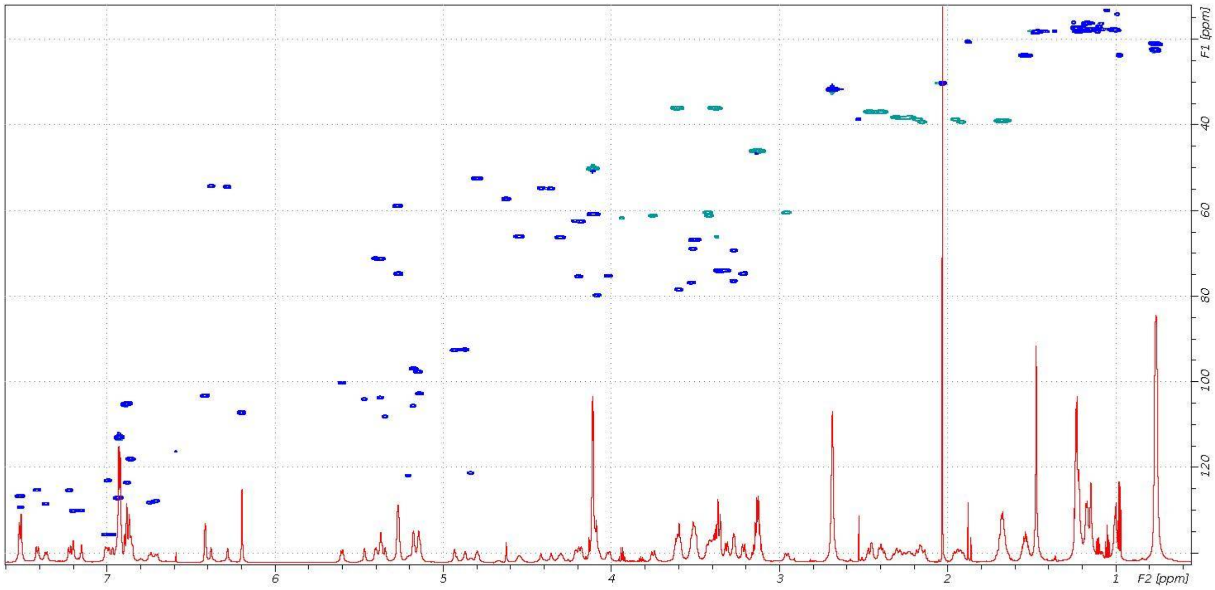

Figure S12. 4k, ^{1}H-^{13}C correlation, 288K, D2O, 700 MHz ^{1}H-NMR Spectrometer.

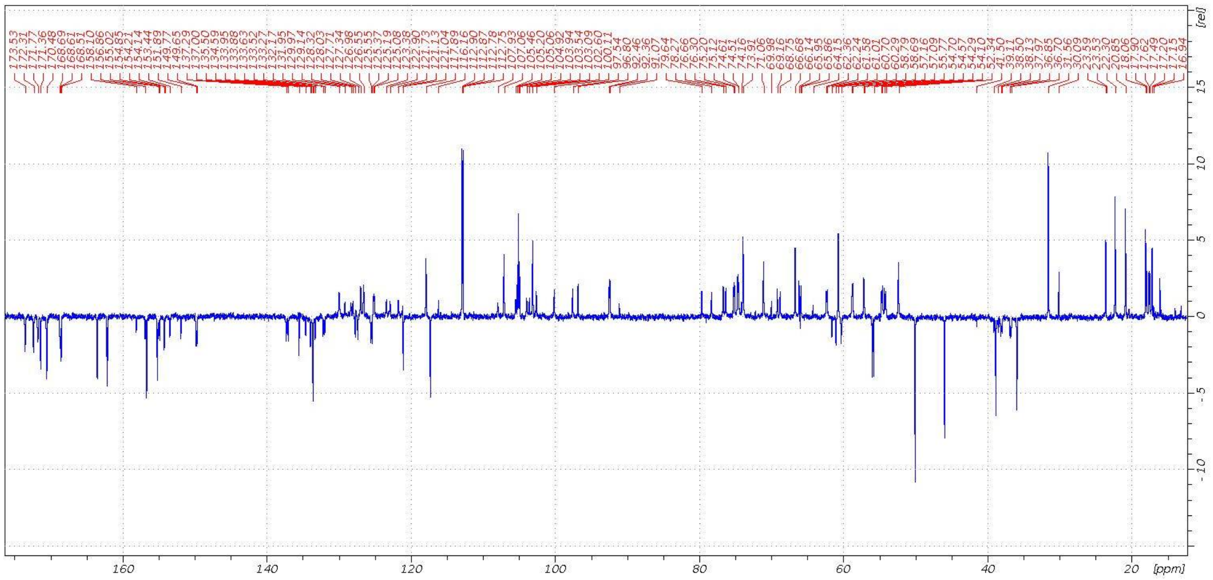

Figure S13. 4k, ^{13}C- NMR, 288K, D2O, 700 MHz.

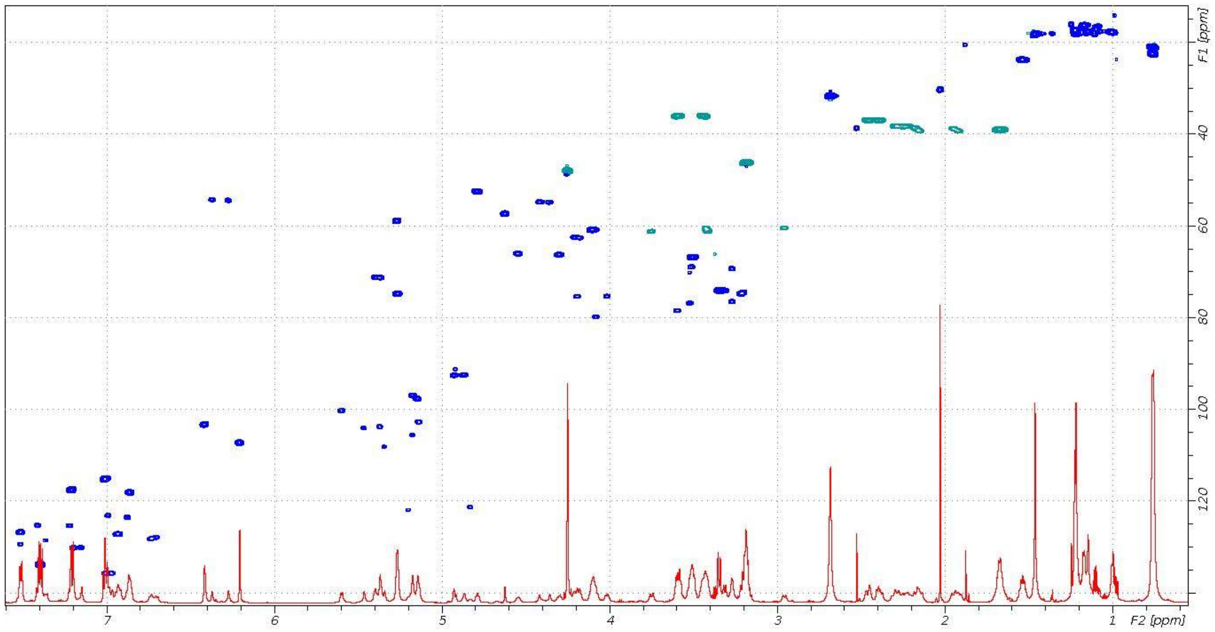

Figure S14. 4l, ^{1}H-^{13}C correlation, 288K, D2O, 700 MHz ^{1}H-NMR Spectrometer.

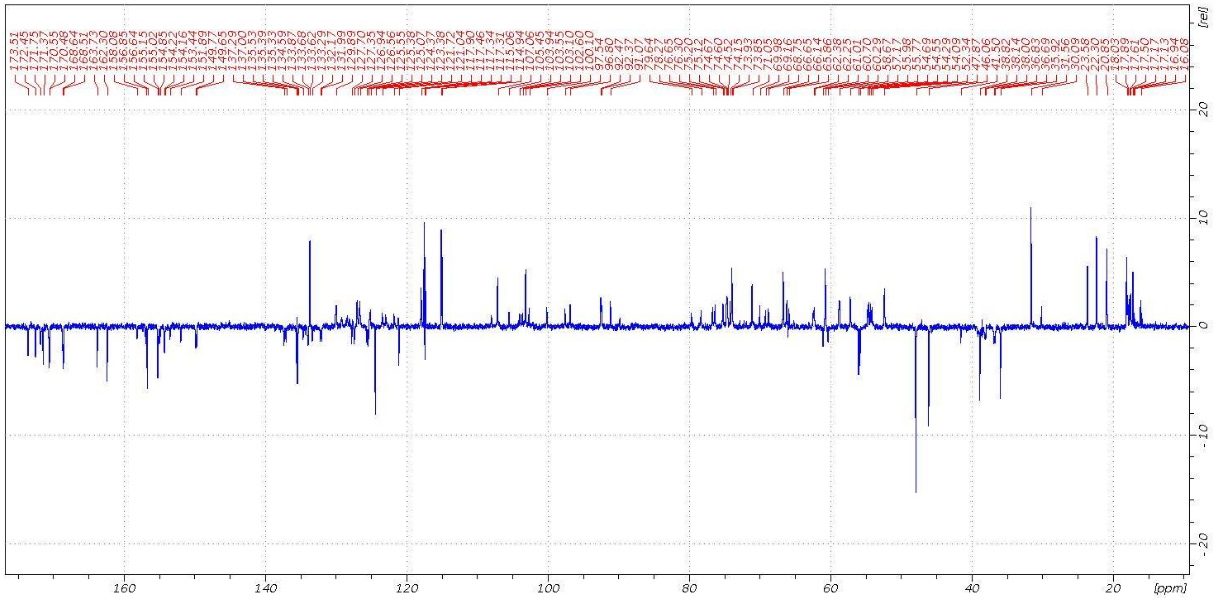

Figure S15. 4l, ^{13}C- NMR, 288K, D2O, 700 MHz.

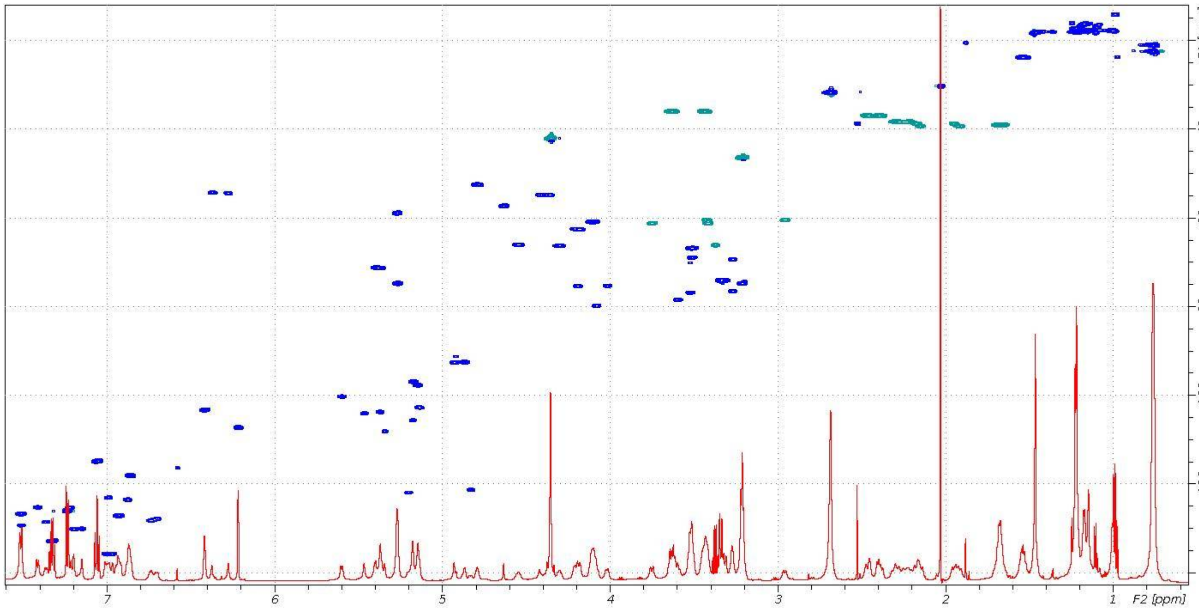

Figure S16. 4m, ^{1}H-^{13}C correlation, 288K, D2O, 700 MHz ^{1}H-NMR Spectrometer.

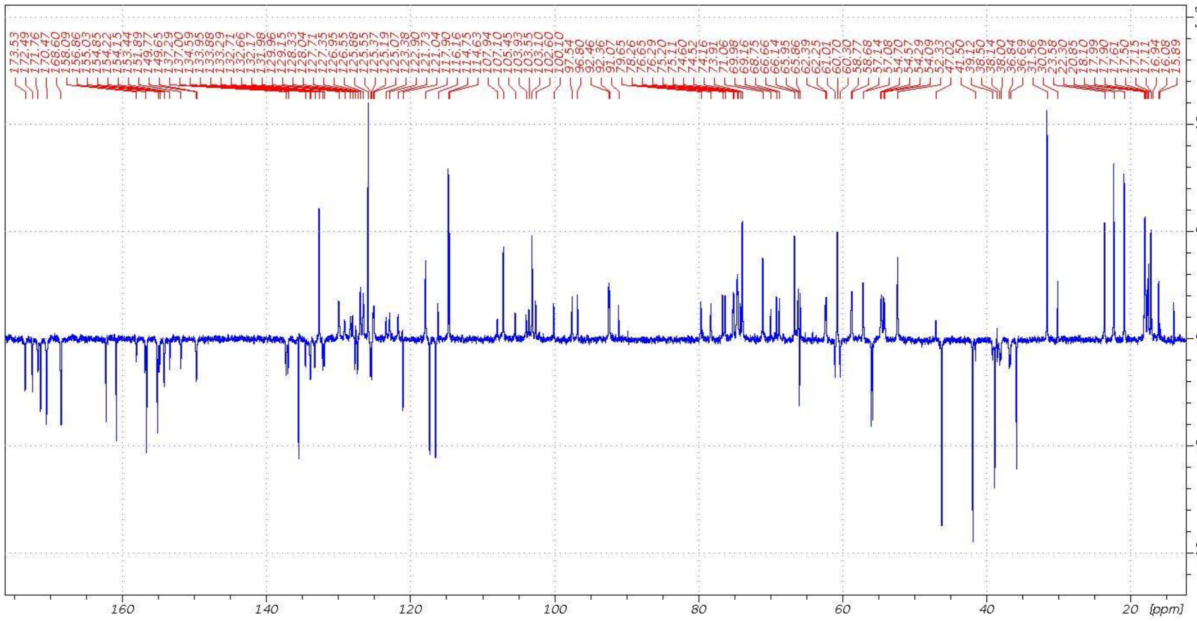

Figure S17. 4m, ^{13}C- NMR, 288K, D2O, 700 MHz.

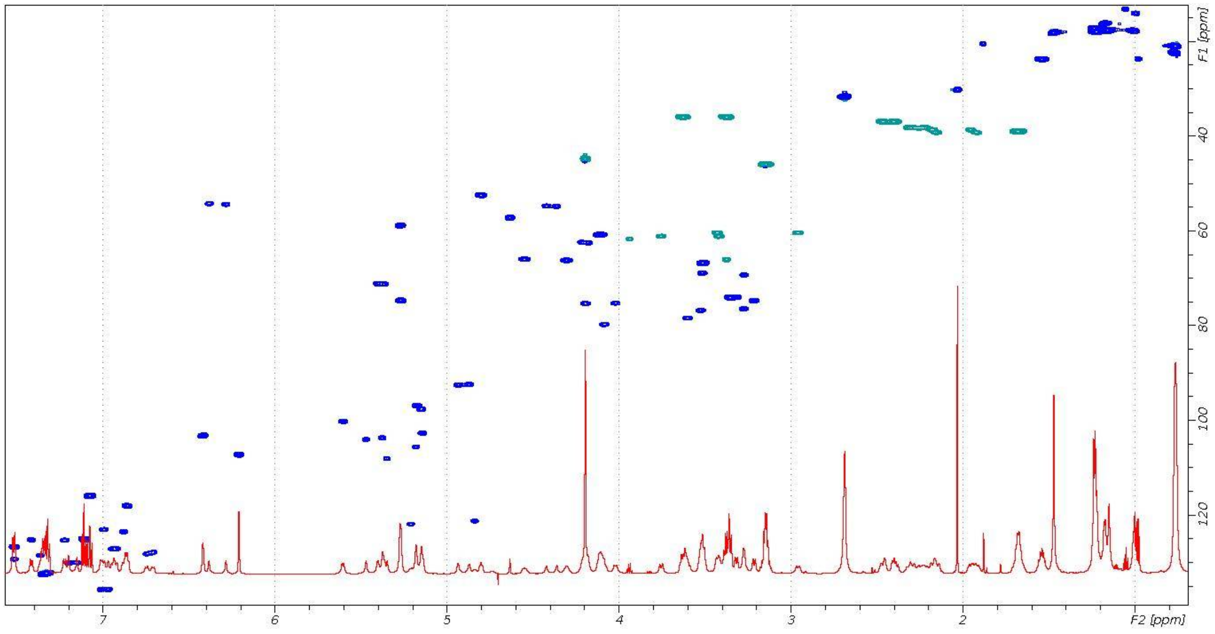

Figure S18. 4p, ^{1}H-^{13}C correlation, 288K, D2O, 700 MHz ^{1}H-NMR Spectrometer.

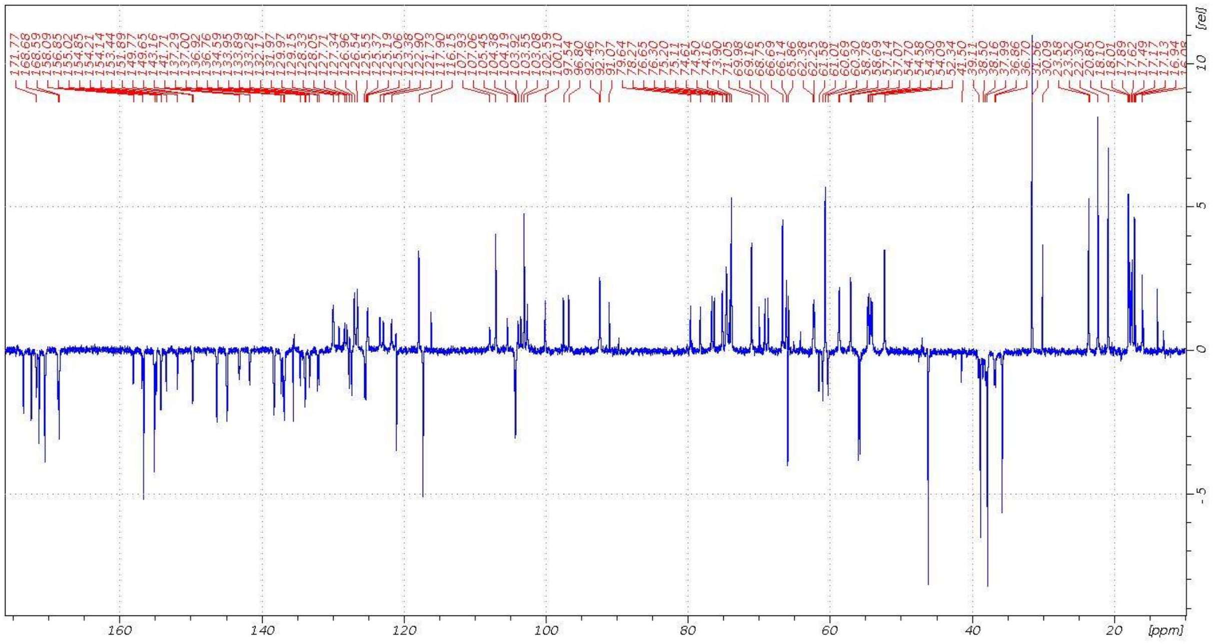

Figure S19. 4p, ^{13}C- NMR, 288K, D2O, 700 MHz.

Supplementary Data

Table S1. C-terminal tags in eremomycin derivatives, ^{13}C assignments (/^{1}H shifts, in case of ^{13}C overlap). Multiplicity of ^{13}C signals due to ^{19}F spin–spin couplings are given as d (doublet) or t (triplet).

Compound	t1	t2	t3	t4	t5	t6	t7	t8	t9	t10
4e	117.28	160.97(d)	115.80(d)	132.07	124.96	132.27(d)	44.68	45.84	35.85	-
4f	117.33(d)	160.92(d)	115.75(d)	132.01	124.89	132.21(d)	44.53/4.13	44.57/2.91	24.92	36.38
4g	132.49(d)	(116.56) [a]	162.44(d)	(116.45)[a]	131.03	125.61	50.40	45.75	35.94	-
4h	126.22	131.97(d)	116.0(d)	163.07(d)	116.0(d)	131.97(d)	50.31	45.63	38.83	-
4j	106.41(t)	161.21(d)	111.78(d)	132.86(t)	111.78(d)	161.21(d)	38.26	45.92	35.73	-
4k	133.57(t)	112.83(d)	162.81(d)	105.06(t)	162.81(d)	112.83(d)	50.04	45.95	35.91	-
4l	(124.37)	(135.36)	117.37(d)	163.01(d)	114.98(d)	133.65(d)	47.87	46.05	35.92	-
4m	116.51(d)	135.52	125.88	132.69(d)	114.70(d)	161.56(d)	41.89	46.19	35.80	-
4p	104.29(t)	145.62(d)	137.54(dt)	142.43(dt)	137.54(dt)	145.62(d)	37.82	46.16	35.77	-

Table S2. Partial ^{1}H/^{13}C NMR signal assignment of compound **4e** including several fingerprint assignments.

Assignment	^{13}C-shift (ppm)	^{1}H-shift (ppm)	^{1}H-shift (ppm)
y8	173.53	-	
y7	172.37	-	
y4	171.76	-	
y5	171.36	-	
y3	170.53	-	
y1	168.63	-	
y6	168.52	-	
y2	168.51	-	
t2	160.97(d)	-	
7e	156.65	-	
7c	155.15	-	
7a	135.53	-	
t6	132.27(d)	7.33	
t4	132.07	7.32	
6b	129.18	7.513	
6b*	128.36	7.361	
6f -6f*	128.03	6.740	
6f-6f*	127.72	6.707	
t5	124.96	7.109	
6c	123.39	6.879	
6c*	122.91	6.993	
6e	121.75	5.206	
6e*	121.13	4.836	
7b	117.28	-	
t1	117.28	-	
t3	115.80(d)	7.073	
7f	107.11	6.206	
7d	103.1	6.414	
g1*	102.57	5.138	
g1	100.08	5.601	
f1	97.52	5.147	
f1	96.77	5.176	
e1	92.47	4.931	
e1	92.33	4.868	
g2 *	79.64	4.081	
g2	78.29	3.596	
g3	76.67	3.520	
g5	76.31	3.270	
g3*	75.20	4.190	
g5*	75.11	4.016	
z6	74.56	5.264	
g4*	73.907	3.349	
z2	71.07	5.39	5.37
g4	68.767	3.512	
x6	62.30	4.21	4.18
g6	61.00	3.752	
g6	61.00	3.413	
x1	60.65	4.117	
g6*	60.31	2.956	
x2	58.75	5.266	
x7	57.11	4.63	
x5	54.74	4.396	
x4*	54.40	6.279	
x4	54.10	6.383	
x3	52.34	4.796	
t8	45.84	3.142	
t7	44.68	4.192	
1a	38.815	1.675	
f2-f2*	38.517	1.949	
e2-e2*	38.099	2.280	
3a	36.979	2.428	
t9	35.85	3.621	3.371
g6*	30.24	3.425	
1e	30.113	2.028	
1b	23.551	1.536	
1d	22.239	0.763	
1c	20.868	0.762	

Table S3. Characterization of the strains used in the study.

Strains	Strain characterization	Source
Staphylococcus aureus ATCC 29213	Quality control strain	Russian Scientific Research Institute of Hematology and Transfusiology
Staphylococcus aureus 3797	Glycopeptide intermediate	GISA HIP-5836 New Jersey, Lepetit Research Center (LePetit Group, Biosearch S.p.A., Varese, Italy)
Staphylococcus aureus 3798	Glycopeptide intermediate	GISA HIP-5827 Michigan, Lepetit Research Center (LePetit Group, Biosearch S.p.A., Varese, Italy)
Staphylococcus aureus R-2	Clinical isolate, MRSA, resistance to Doripenem	Multicenter studies of Doripenem
Staphylococcus haemoliticus 602	Clinical isolate	Lepetit Research Center (LePetit Group, Biosearch S.p.A., Varese, Italy)
Streptococcus pneumoniae ATCC 6305	Quality control strain	Russian Scientific Research Institute of Hematology and Transfusiology
Enterococcus faecium 4	Clinical isolate, Vancomycin- susceptible.	Russian Scientific Research Institute of Hematology and Transfusiology
Enterococcus. faecium 2	Clinical isolate, Vancomycin- resistance (Van E)	Russian Scientific Research Institute of Hematology and Transfusiology
Enterococcus faecium 3576	Clinical isolate, Vancomycin- resistance (VanA)	N.N.Blokhin Russian Cancer Research Centre
Enterococcus faecalis 9	Clinical isolate, Vancomycin- resistance (VanB)	Russian Scientific Research Institute of Hematology and Transfusiology
Enterococcus faecalis 583	Clinical isolate, Vancomycin- resistance (Van E)	N.N.Blokhin Russian Cancer Research Centre
Enterococcus faecalis 559	Clinical isolate, Vancomycin- susceptible.	Lepetit Research Center (LePetit Group, Biosearch S.p.A., Varese, Italy)
Enterococcus. gallinarum ВП 4147	Clinical isolate, Vancomycin- resistance	N.N.Blokhin Russian Cancer Research Centre

Table S4. Antibacterial activity of vancomycin and derivatives 4a–q against sensitive Gram-positive bacteria.

Compound	m	n	R	Strain/MIC (Minimum inhibitory concentration, µg/ml)*					
				S.aureus ATCC 29213	*S.aureus* R-2	*S.haemoliticus* 602	*S.pneumoniae* ATCC 6305	*E. faecium* 4	*E. faecalis* 559
**Van**	-	-	-	0.25-**0.5**	1	2	0,25	4	2
**4a**	2	1	-	0.25	0.03	0.5	0.25	0.25-**0.5**	0.25
**4b**	2	2	-	0.5	0.06	0.5	0.01	0.5	0.5
**4c**	2	1	-4-CH_3	0.25	0.25	0.25	0.01	0.25	0.25
**4d**	2	1	-4-OCH_3	0.03	0.06	0.25	0.01	0.25	0.5
**4e**	2	1	-2-F	0.125	**0.03**-0.06	0.125	0.01	0.25	0.125
**4f**	3	1	-2-F	0.125	0.03	0.25-**0.5**	0.03	0.25	**0.25**-0.5
**4g**	2	1	-3-F	0.03	0.06	0.5	0.01	0.25	0.25-**0.5**
**4h**	2	1	-4-F	0.25	0.06	0.25	0.01	0.25	0.125
**4i**	2	1	-4-Cl	0.125-**0.25**	0.25	0.25	00.03	0.25	0.5
**4j**	2	1	-2,6-F_2	**0.03**-0.06	0.06	0.125	0.03	**0.25**-0.5	0.25
**4k**	2	1	-3,5-F_2	0.25	0.125-**0.25**	0.125	0.01	0.5	0.5
**4l**	2	1	-2-Cl,-4-F	0.125	0.25	0.125	0.03-**0.06**	0.25	1
**4m**	2	1	-2-Cl,-6-F	0.25	0.125-**0.25**	0.125	0.06	0.5	0.5-**1**
**4n**	2	1	-2,4-Cl_2	0.5	0.25-**0.5**	0.25	0.03-**0.06**	1	1
**4o**	2	1	-3,4-Cl_2	1	1	0.5	0.5	1	2
**4p**	2	1	-1,2,3,4,5-F_5	0.25	0.25	**0.25**-0.5	0.01	1	1
**4q**	2	1	-4-CF_3	1	2	0.5	0.01	1	2

*MICs are presented as modal values for 3–5 independently replicated MIC tests for each strain and for the antibiotics. In the range of the obtained MIC values, the obtained modal values are highlighted in bold.

Table S5. Antibacterial activity of vancomycin and derivatives 4a–q against resistant Gram-positive bacteria.

Compound	m	n	R	Strain/MIC (minimum inhibitory concentration; µg/ml)						
				S.aureus 3797	*S.aureus* 3798	*E. faecium* 2	*E. faecium* 3576	*E. faecalis* 9	*E. faecalis* 583	*E. gallinarum* ВП 4147
**Van**	-	-	-	8	8	8	>32	8	32	8
**4a**	2	1	-	0.5	0.5-**1**	1	**1**-2	0.06	0.5	1
**4b**	2	2	-	1	2	1	2	0.06	0.5	1
**4c**	2	1	-4-CH_3	0.5	1	1	1	0.03	**1**-2	0.5
**4d**	2	1	-4-OCH_3	0.5	0.5-**1**	0.5	0.5-**1**	0.03	0.5	1
**4e**	2	1	-2-F	0.5	1	0.5	**0.5**-1	**0.03**-0.06	0.25-**0.5**	0.5
**4f**	3	1	-2-F	0.5	2	1	1	0.06	1	0.5
**4g**	2	1	-3-F	1	2	0.5	1	0.03	**0.5**-1	1
**4h**	2	1	-4-F	0.25	0.5	0.5	2	0.03	**0.5**-1	**0.5**-1
**4i**	2	1	-4-Cl	0.25-**0.5**	1	0.5	1	0.06	0.5-**1**	0.5
**4j**	2	1	-2,6-F_2	0.5	1	0.5-**1**	1	0.03	0.5	1
**4k**	2	1	-3,5-F_2	0.5	1	1	1	0.06	1	1
**4l**	2	1	-2-Cl,-4-F	2	1	1	2	0.25	1	1
**4m**	2	1	-2-Cl,-6-F	2	2	1	2	0.25-**0.5**	1	2
**4n**	2	1	-2,4-Cl_2	1	2	2	2	0.25	2	2
**4o**	2	1	-3,4-Cl_2	2	1-**2**	2	2	**0.25**-0.5	2	2
**4p**	2	1	-1,2,3,4,5-F_5	1-**2**	4	1	4	0.25	1	2
**4q**	2	1	-4-CF_3	2	2	1-**2**	4	0.25	4	4

*MICs are presented as modal values for 3–5 independently replicate MIC tests for each strain and for the antibiotics. In the range of the obtained MIC values, the obtained modal values are highlighted in bold.
